# Associated factors of diabetic retinopathy by artificial intelligence evaluation of fundus images in Japan

**DOI:** 10.1038/s41598-023-47270-x

**Published:** 2023-11-13

**Authors:** Koji Komatsu, Kei Sano, Kota Fukai, Ryo Nakagawa, Takashi Nakagawa, Masayuki Tatemichi, Tadashi Nakano

**Affiliations:** 1 Department of Ophthalmology, The Jikei University School of Medicine, 3-25-8 Nishi-Shimbashi, Minato-Ku, Tokyo, 105-8461 Japan; 2 Department of Preventive Medicine, School of Medicine, Tokai University, Kanagawa, Japan; 3 Omiya City Clinic, Saitama, Japan

**Keywords:** Medical research, Risk factors

## Abstract

This cross-sectional study aimed to investigate the promoting and inhibitory factors of diabetic retinopathy (DR) according to diabetes mellitus (DM) stage using standardized evaluation of fundus images by artificial intelligence (AI). A total of 30,167 participants underwent blood and fundus examinations at a health screening facility in Japan (2015–2016). Fundus photographs were screened by the AI software, RetCAD and DR scores (DRSs) were quantified. The presence of DR was determined by setting two cut-off values prioritizing sensitivity or specificity. DM was defined as four stages (no DM: DM0; advanced DM: DM3) based on treatment history and hemoglobin A1c (HbA1c) levels. Associated factors of DR were identified using logistic regression analysis. For cutoff values, multivariate analysis revealed age, sex, systolic blood pressure (SBP), smoking, urinary protein, and HbA1c level as positively associated with the risk of DR among all DM stages. In addition to glycemic control, SBP and Fibrosis-4 index might act as promoting factors for DR at all or an earlier DM stage. T-Bil, cholinesterase, and T-cho level might be protective factors at an advanced DM stage.

## Introduction

The International Diabetes Federation estimated the global population with diabetes mellitus (DM) as 463 million in 2019 and 700 million by 2045^1^. In Japan, there are 10 million patients with DM and 3 million with diabetic retinopathy (DR), which is the third leading cause of visual disorders (12.8%)^2^.

The risk and protective factors of DR have been intensively studied. However, as long-term glycemic control is an extremely determinant factor, other factors or biomarkers show largely inconsistent results according to a Cochrane Review^3^. In a recent quantification analysis of risk factors for DR progression, the average hemoglobin A1c (HbA1c) level had the strongest impact on DR progression, followed by average systolic blood pressure (SBP) control and total cholesterol (T-Cho) level^4^. A Mendelian randomization study suggested that higher body mass index (BMI), higher waist-to-hip ratio (WHR), and smoking were likely to be causal factors in DR development, whereas genetically higher hip circumstance was associated with a lower risk of DR^5^. In a meta-analysis assessing the risk factors for DR, abdominal obesity, assessed as the WHR, was associated with DR in patients with DM; however, no correlation was found between abdominal obesity and varying degrees of DR^6^. A recent nutrient review demonstrated that higher intake of fruits, vegetables, dietary fiber, fish, oleic acid, and tea and a Mediterranean diet had a protective effect against DR; conversely, high intake of diet soda, calories, rice, and choline was associated with a higher risk of DR^7^.

We further considered two major reasons for the lack of clear risk factors for DR other than glycemic control^8^. First, this may be due to inconsistent diagnosing. Critical issues have been highlighted in identifying the risk factors for DR in the general population. Misclassifications in fundus evaluation can occur because of the experience and skills of the reading ophthalmologists. Kawasaki et al.^9^ reported a kappa value of 0.56, which was determined by a local ophthalmologist and two retinal specialists, in a study of 1,221 participants. In a study of 1,806 patients by Hashimoto et al.^10^, two ophthalmologists and, in the case of a split decision, three retinal specialists, made the classification decision. Consequently, outcomes are unstable. Second, as a long duration of diabetes is critical^3^, risk and protective factors may vary based on the diabetic stage.

In recent years, various artificial intelligence (AI) medical devices using deep learning have become popular, and Food and Drug Administration-approved DR screening AI software has been utilized. AI evaluation of posterior polar fundus photographs is not inferior to wide-angle fundus photograph evaluation by a retinal specialist^11^. The AI software, RetCAD, achieved an area under the receiver operating characteristic (ROC) curve (AUC) of 95.1% for DR detection (severity not determined), with a sensitivity of 90.1% and specificity of 90.6%^12^. This evaluation takes into consideration a severity level at which moderate to severe non-proliferative diabetic retinopathy (NPDR) can be reliably detected, and there is also potential for identifying mild NPDR. This assessment aligns with the criteria set forth by the International Clinical Diabetic Retinopathy Disease Severity Scale^13^. Therefore, in this study, we investigated the risk factors for DR by establishing the DR diagnosis using AI and estimating the associated factors for each diabetic stage using data from a large number of health screening examinations.

## Results

Table 1 shows the characteristics of the participants according to DM stage and DRS with a cutoff value of 20. A high DRS was observed in 3.6% (n = 1,076) of participants. Additionally, 3.0%, 5.0%, 13%, and 23.6% of participants were classified as DM0, DM1, DM2, and DM3, respectively. As the DM stage increased, the percentage of high DRS also increased. When compared by sex, there was a trend toward a higher percentage of males with DM2 and DM3 being treated for DM.

**Table 1:** Participants’ characteristics according to DM stage and DRS (cutoff value, 20).

		DM0		DM1		DM2		DM3		Total	
		DRS < 20	DRS ≥ 20	DRS < 20	DRS ≥ 20	DRS < 20	DRS ≥ 20	DRS < 20	DRS ≥ 20	DRS < 20	DRS ≥ 20
All	n (%)	27,517 (97.0)	844 (3.0)	530 (95.0)	28 (5.0)	743 (87.0)	111 (13.0)	301 (76.4)	93 (23.6)	29,091 (96.4)	1076 (3.6)
	DRS	14.1 ± 1.3	26.8 ± 8.3	14.3 ± 1.4	36.4 ± 14.9	14.7 ± 1.5	35.4 ± 15.4	14.7 ± 1.5	41.8 ± 16.9	14.1 ± 1.3	29.2 ± 11.5
	Age	49.3 ± 8.5	52 ± 8.9	54.7 ± 8.2	53 ± 10.6	56.3 ± 7.9	55.5 ± 8.8	53.7 ± 8	52.8 ± 8	49.6 ± 8.6	52.5 ± 8.9
	HbA1c	5.5 ± 0.3	5.6 ± 0.3	7.2 ± 1.1	8 ± 1.9	6.6 ± 0.5	6.7 ± 0.4	8.6 ± 1.2	8.9 ± 1.3	5.6 ± 0.5	6 ± 1.2
Males	n (%)	15,258 (96.7)	525 (3.3)	383 (95.3)	19 (4.7)	597 (86.1)	96 (13.9)	235 (74.8)	79 (25.2)	16,473 (95.8)	719 (4.2)
	DRS	14.2 ± 1.3	26.8 ± 8.5	14.3 ± 1.4	36.5 ± 14.1	14.7 ± 1.5	36.4 ± 15.9	14.8 ± 1.6	42.1 ± 16.6	14.2 ± 1.4	30 ± 12.3
	Age	49.3 ± 8.7	52 ± 8.8	54.3 ± 8.1	51.2 ± 9.3	56.2 ± 8	55.6 ± 8.8	53.5 ± 7.8	52.8 ± 8	49.8 ± 8.8	52.5 ± 8.8
	HbA1c	5.5 ± 0.3	5.6 ± 0.3	7.1 ± 1	7.8 ± 1.7	6.6 ± 0.5	6.7 ± 0.4	8.6 ± 1.3	8.9 ± 1.3	5.7 ± 0.6	6.2 ± 1.2
Females	n (%)	12,259 (97.5)	319 (2.5)	147 (94.2)	9 (5.8)	146 (90.7)	15 (9.3)	66 (82.5)	14 (17.5)	12,618 (97.2)	357 (2.8)
	DRS	14 ± 1.3	26.8 ± 8	14.4 ± 1.4	36.4 ± 17.6	14.6 ± 1.3	29.2 ± 9.5	14.4 ± 1.4	39.6 ± 19.3	14 ± 1.3	27.6 ± 9.5
	Age	49.2 ± 8.4	52 ± 9.1	55.6 ± 8.5	56.7 ± 12.7	56.7 ± 7.3	55.4 ± 9.2	54.5 ± 9	53.1 ± 8	49.3 ± 8.4	52.3 ± 9.2
	HbA1c	5.5 ± 0.3	5.6 ± 0.4	7.3 ± 1.3	8.4 ± 2.4	6.6 ± 0.5	6.6 ± 0.5	8.4 ± 1.2	8.8 ± 1.1	5.6 ± 0.5	5.8 ± 0.9

Continuous variables are presented as mean ± standard deviation.

Abbreviations: *DM* Diabetes mellitus, *DRS* Diabetic retinopathy score, *Hb* Hemoglobin.

Table 2 shows the characteristics of the participants according to DM stage and DRS with a cutoff value of 50. Because of the higher DR criterion, high DRS was observed in 0.3% of participants. The percentage of high DRS similarly became higher as the DM stage increased. Moreover, the percentage of DM0 was 3.3% with a cutoff value of 20 (Table 1), but was 0.1% with a cutoff value of 50 (Table 2). This indicates improvement in the accuracy of determination.

**Table 2:** Participants’ characteristics according to DM stage and DRS (cutoff value, 50).

		DM0		DM1		DM2		DM3		Total	
		DRS < 50	DRS ≥ 50	DRS < 50	DRS ≥ 50	DRS < 50	DRS ≥ 50	DRS < 50	DRS ≥ 50	DRS < 50	DRS ≥ 50
All	n (%)	28,330 (99.9)	31 (0.1)	552 (98.9)	6 (1.1)	830 (97.2)	24 (2.8)	358 (90.9)	36 (9.1)	30,070 (99.7)	97 (0.3)
	DRS	14.4 ± 2.6	55.8 ± 4.6	15 ± 3.9	59.2 ± 6.8	16.1 ± 5.1	60.6 ± 7	17.1 ± 6.6	60.3 ± 8.8	14.5 ± 2.8	58.9 ± 7.3
	Age	49.3 ± 8.6	54.7 ± 7	54.6 ± 8.3	47.8 ± 5.4	56.3 ± 8	53.9 ± 9.4	53.6 ± 8	52.8 ± 8.1	49.7 ± 8.7	53.4 ± 8
	HbA1c	5.5 ± 0.3	5.7 ± 0.3	7.2 ± 1.1	10.3 ± 2	6.6 ± 0.5	6.7 ± 0.5	8.6 ± 1.2	9 ± 1.4	5.6 ± 0.6	7.4 ± 1.9
Males	n (%)	15,761 (99.9)	22 (0.1)	398 (99.0)	4 (1.0)	670 (96.7)	23 (3.3)	282 (89.8)	32 (10.2)	17,111 (99.5)	81 (0.5)
	DRS	14.5 ± 2.6	55.5 ± 3.9	15 ± 3.9	57.3 ± 7.1	16.2 ± 5.3	60.9 ± 6.9	17.4 ± 6.7	59.7 ± 8	14.7 ± 3	58.8 ± 7
	Age	49.4 ± 8.7	54.7 ± 6	54.2 ± 8.2	50 ± 4.7	56.2 ± 8.1	53.2 ± 8.9	53.4 ± 7.8	52.6 ± 8.2	49.9 ± 8.8	53.2 ± 7.7
	HbA1c	5.5 ± 0.3	5.7 ± 0.3	7.1 ± 1	9.8 ± 2.3	6.6 ± 0.5	6.7 ± 0.5	8.7 ± 1.2	9 ± 1.4	5.7 ± 0.6	7.5 ± 1.8
Females	n (%)	12,569 (99.9)	9 (0.1)	154 (98.7)	2 (1.3)	160 (99.4)	1 (0.6)	76 (95.0)	4 (5.0)	12,959 (99.9)	16 (0.01)
	DRS	14.3 ± 2.4	56.4 ± 6.2	15 ± 3.9	63 ± 6.1	15.7 ± 4.4	53 ± 0	16.4 ± 6	65 ± 14.7	14.4 ± 2.5	59.2 ± 9.2
	Age	49.2 ± 8.4	54.7 ± 9.4	55.8 ± 8.7	43.5 ± 4.9	56.5 ± 7.4	70 ± 0	54.3 ± 8.9	54.3 ± 7.6	49.4 ± 8.5	54.1 ± 9.6
	HbA1c	5.5 ± 0.3	5.8 ± 0.3	7.3 ± 1.3	11.5 ± 0.6	6.6 ± 0.5	6.4 ± 0	8.5 ± 1.2	8.4 ± 1.2	5.6 ± 0.5	7.2 ± 2.1

Continuous variables are presented as mean ± standard deviation.

Abbreviations: *DM* Diabetes mellitus, *DRS* Diabetic retinopathy score, *Hb* Hemoglobin.

Table 3 provides the results of the logistic analyses with cutoff values of 20 (sensitivity, 100%) and 50 (specificity, 100%) for the identification of the risk factors for DR. Variables were selected for logistic analysis based on significant group differences on ANCOVA (Supplemental Table S1) and clinical importance. With a cutoff value of 20, SBP (odds ratio [OR] 1.01; 95% confidence interval [CI] 1.01–1.02), smoking (OR 1.37; 95% CI 1.18–1.60), urinary protein level (OR 1.69; 95% CI 1.17–2.44), and HbA1c level (OR 1.67; 95% CI 1.57–1.78) were significantly and positively associated with a high risk of DR. For T-Cho classified by tertile, higher T-Cho level (> 240) (OR 0.82; 95% CI 0.68–0.98) was negatively associated with a high risk of DR. As T-Cho level increased, the risk of DR decreased (p_trend_ = 0.03). With a cutoff value of 50, SBP (OR 1.03; 95% CI 1.02–1.04), smoking (OR 1.97; 95% CI 1.22–3.18), urinary protein level (OR 3.00; 95% CI 1.45–6.20), and HbA1c level (OR 2.44; 95% CI 2.17–2.75) were positively associated with a high risk of DR. In addition, for ChE classified by tertile, high ChE level (> 361) (OR 0.52; 95% CI 0.29–0.93) was a protective factor against DR, and as ChE increased the risk of DR decreased (p_trend_ = 0.03). Similarly, higher T-Cho level (> 240) (OR 0.33; 95% CI 0.15–0.73) was negatively associated with DR, and as T-Cho increased the risk of DR decreased (p_trend_ = 0.002).

**Table 3:** Risk factors for high DRS.

		DRS cutoff value = 20			DRS cutoff value = 50		
		OR	95%CI		OR	95%CI	
Age		1.02	1.01	1.03	1.03	1.00	1.07
Sex, male		1.20	1.04	1.40	2.26	1.22	4.18
SBP		1.01	1.01	1.02	1.03	1.02	1.04
T-Bil		1.06	0.90	1.25	0.53	0.27	1.07
ChE	< 303	Reference			Reference		
	303–361	0.94	0.80	1.11	0.69	0.39	1.22
	> 361	0.87	0.73	1.03	0.52	0.29	0.93
	p for trend	0.10			0.03		
FIB-4 index		1.09	0.96	1.25	0.96	0.64	1.45
T-Cho	< 220	Reference			Reference		
	220–240	0.91	0.77	1.08	0.53	0.27	1.06
	> 240	0.82	0.68	0.98	0.33	0.15	0.73
	p for trend	0.03			0.00		
eGFR		1.00	0.99	1.002	0.99	0.98	1.01
Smoking		1.37	1.18	1.60	1.97	1.22	3.18
Urinary protein		1.69	1.17	2.44	3.00	1.45	6.20
HbA1c		1.67	1.57	1.78	2.44	2.17	2.75

P for trend was calculated by converting each class of ChE or T-Cho into continuous variables.

Abbreviations: *DRS* Diabetic retinopathy score, *OR* Odds ratios, *CI* Confident interval, *SBP* Systolic blood pressure, *T-Bil* Total bilirubin, *ChE* Cholinesterase, *T-Cho* Total cholesterol, *eGFR* Estimated glomerular filtration rate, *Hb* Hemoglobin.

Finally, logistic analysis was performed to identify the risk factors for DRS according to DM stage in addition to glycemic control using a cutoff value of 20. HbA1c-adjusted ORs are shown in Table 4. For DM1, SBP (OR 1.02; 95% CI 0.998–1.04) and FIB-4 score (OR 2.04; 95% CI 1.10–3.81) were significantly and positively associated with a high risk of DR. For DM2, SBP (OR 1.02; 95% CI 1.01–1.03) was positively associated with a high risk of DR, while the T-Bil level (OR 0.34; 95% CI 0.17–0.68) and the ChE level (OR 0.99; 95% CI 0.99–0.0.997) were negatively associated with DR. For DM3, SBP (OR 1.02; 95% CI 1.00–1.03) was positively associated with a high risk of DR, while a T-Cho level of 220–240 mg/dL (OR 0.16; 95% CI 0.04–0.68) and high T-Cho level (> 240 mg/dL) (OR 0.44; 95% CI 0.20–0.997) were negatively associated with a high risk of DR.

**Table 4:** Risk factors for high DRS stratified by DM stage and adjusted for HbA1c.

		DM1			DM2			DM3		
		OR	95% CI		OR	95% CI		OR	95% CI	
Age		0.97	0.91	1.03	0.96	0.94	0.99	1.01	0.97	1.05
Sex		0.79	0.30	2.08	1.65	0.89	3.07	1.35	0.67	2.71
SBP		1.02	0.998	1.04	1.02	1.01	1.03	1.02	1.00	1.04
T-Bil		0.70	0.20	2.42	0.34	0.17	0.68	0.90	0.42	1.91
ChE		0.998	0.99	1.004	0.99	0.99	0.997	0.996	0.99	0.999
FIB4		2.04	1.10	3.81	1.17	0.89	1.54	0.61	0.36	1.05
T-Cho	< 220	1.00			1.00			1.00		
	220–240	0.65	0.23	1.84	0.92	0.46	1.85	0.16	0.04	0.68
	> 240	0.25	0.06	1.04	1.70	0.86	3.38	0.44	0.20	0.997
	p for trend	0.052			0.22			0.01		
eGRF		1.002	0.98	1.03	0.997	0.98	1.01	1.01	0.99	1.02
Smoking		0.92	0.36	2.36	0.80	0.49	1.30	1.41	0.82	2.42
Urine-protein		1.57	0.27	9.14	2.03	0.90	4.59	1.33	0.60	2.94

p for trend was calculated by converting the classes of T-Cho into a continuous variable.

Abbreviations: DRS = diabetic retinopathy score, DM = diabetes mellitus, HbA1c = hemoglobin A1c, OR = odds ratio, CI = confidence interval, SBP = systolic blood pressure, T-Bil = total bilirubin, ChE = cholinesterase, FIB4 = fibrosis 4 score, T-Cho = total cholesterol, eGFR = estimated glomerular filtration rate.

## Discussion

The present study is the first to identify factors associated with DR on health screening examinations using quantitative evaluation of fundus images by AI. In this study, two DRS cutoff values were examined (20 and 50), corresponding to 100% sensitivity and 100% specificity, respectively. This approach covered exploratory risk extraction with 100% sensitivity; however, at 100% sensitivity lesions other than DR were included. Therefore, a specificity of 100% was used to confirm whether it was a DR-specific factor. Thus, in addition to glucose control, which was a determining factor, we found that SBP and smoking habit were probable risk factors. Furthermore, we found several potential risk factors, such as urinary protein level, and protective factors, T-Bil level, FIB-4 index score, and ChE level, in some stages of DM.

When prioritizing sensitivity for DR by AI diagnosis, 3% of participants showed high DRS in addition to DR, and changes related to arteriosclerosis might have been included. For example, the AI software used in this study tended to falsely detect retinal hemorrhage due to central or branch retinal vein occlusion as DR. Thus, only SBP and smoking were significantly associated with DR. When prioritizing specificity for DR by AI diagnosis, more specific factors for DR were selected. These results indicate that SBP and smoking are consistently significant risk factors for DR progression.

A review by Sharma et al.^14^ reported that the main pathophysiological changes in DR caused by chronic hyperglycemia included the following: (1) local ischemia and (2) basement membrane dysfunction and thickening and pericyte depletion. The major metabolic abnormalities induced by hyperglycemia involve increased glucose flux through the activation of the polyol, hexosamine, protein kinase C, and angiotensin II pathways and the accumulation of advanced glycation end-products, contributing to an imbalance in cellular redox homeostasis. Such vicious cycles cause high levels of reactive oxygen species (ROS) to be produced during oxidative stress, which results in apoptosis.

Regarding SBP, several studies have reported that a 10-mmHg increase in SBP increases the risk of early DR by 10%^3,4^. Thus, atherosclerotic changes, in addition to smoking, damage the microvessels supplying the retina, leading to ischemia, vascular leakage, and central vision loss caused by diabetic macular edema, in all stages of DM.

In the DM1 group, HbA1c level and FIB-4-index score were positively associated with DR. Participants in this group have neglected diabetes or recently high blood glucose levels and are considered to be in the early stage of diabetes. Thus, the risk of DR was dependent on HbA1c level. The FIB-4 index is a marker of liver fibrosis based on metabolic-associated fatty liver disease^15^. The significance of waist circumference as a risk factor for DR in several studies may be due to fatty liver as a liver lesion in early-stage metabolic syndrome. At this stage, inflammatory cytokines and ROS-related factors might be important for the early progression of DR. A recent study demonstrated that a potent nicotinamide adenine dinucleotide phosphate oxidase 4 inhibitor was effective in treating the early pathological events of DR^16^.

In the DM2 group, which included participants who had a treatment history for DM with good glycemic control, SBP was a risk factor for DR. In contrast, the T-Bil level showed a negative association with DR. As a systemic vascular lesion caused by DM, increased SBP seems to be the second step in the progression of DR. Interestingly, several studies have demonstrated that bilirubin has effective antioxidant properties and is a protective agent against diabetes and cardiovascular diseases^17^. Moreover, a meta-analysis found a negative nonlinear association between bilirubin concentration and the risk of diabetic complications^18^. Ding et al.^19^ reported that the T-Bil level predicts an increased risk of severe DR progression. Decreased bilirubin level might be attributed to increased levels of lipopolysaccharide and urobilinogen, which may indicate that the change in bilirubin level is secondary to intestinal flora disorder and/or intestinal barrier destruction. These reports are consistent with our findings, and bilirubin might be a predictive marker for DR in early-stage DM.

In the DM3 group, which included participants with poor glycemic control, the ChE level was negatively associated with DR. This result was similarly observed on multivariate analysis with a cutoff value emphasizing specificity. There are two main types of ChE: acetylcholinesterase and butyrylcholinesterase (BuChE). The BuChE level is assessed during health examinations as a liver function test because it is synthesized mainly in the liver. An elevation in the BuChE level has been reported in participants with diabetes and fatty liver^20^. A meta-analysis by Song et al.^21^ reported that, although there was no overall association between nonalcoholic fatty liver disease (NAFLD) and DR in patients with type 2 diabetes, subgroup analyses suggested that, in China, Korea, and Iran, patients with type 2 diabetes and NAFLD had a lower risk of DR than those without NAFLD. Similarly, in Japan, NAFLD might be negatively associated with DR. As a possible mechanism, BuChE activity is associated with retinal blood flow through the blood-retinal barrier, and it is reduced by 30–50% in the retina of diabetes-induced mice^22^. Elevation of systemic BuChE levels by fatty liver associated with the FIB-4 index score might protect against the onset of DR.

Regarding lipid metabolism, the US Early Treatment Diabetic Retinopathy Study reported that lipid metabolism was facilitative for DR^23^. In contrast, a recent meta-analysis reported that lipid metabolism was associated with DR^24^. However, a Cochrane Review demonstrated no association between T-Cho level and DR^3^. In this previous study, the T-Cho level was partially associated with DR, showing a U-shaped relationship. Further detailed examinations are required to elucidate this issue.

A strength of the present study is that it used AI to standardize fundus evaluation with an ROC of 95.1% (SE = 90.1%, SP = 90.6%) and accuracy of 95.1%^12^. Owing to the large sample size in the present study (33,022 patients), we were able to determine the factors associated with DR according to DM stage. However, the present study has some limitations. First, this study was cross-sectional in nature. Thus, further follow-up studies are needed. Second, the classification of DM was not dependent on DM duration. However, a high risk of DR was well correlated with the DM stage. Third, the evaluation of DRS utilized posterior polar fundus images. It is possible that DR changes in the peripheral retina were overlooked. The introduction of wide-angle fundus photography equipment and updates to AI software are expected in the future. Fourth, there is a possibility that the pre-validation assessment for the study population was very minimal. It will be necessary to consider the best method for pre-validation in the future.

Our study confirms that standardized evaluation of fundus images by AI can be used to identify factors that promote and protect DR from medical examination data and fundus photographs obtained in general health examinations. This approach can make important contributions to solving previous challenges in the evaluation of DR^8^. Studies on DR risk and the protective factors of DR by deploying this method on a nationwide scale are required.

## Materials and methods

### Study setting and data sources

This cross-sectional study was conducted at a health screening center located in the Tokyo metropolitan area of Japan, the Omiya City Clinic. Specifically, 33,022 patients who underwent fundus examination and blood tests between April 2015 and March 2016 were included in this study.

This study was approved by the ethics committees of the Jikei University School of Medicine (31-428(10010)), Tokai University (20R-005), and Omiya City Clinic (No. 20). All studies were conducted in compliance with the tenets of the Declaration of Helsinki. Information was disclosed to participants, who could opt-out, on the Omiya City Clinic webpage.

### Data collection/measurement

Quantification of fundus images and definition of quality cutoff value.

The fundus images were taken using a non-mydriatic digital fundus camera (CR-2 PlusAF; Canon). Both eyes were imaged centered on the macula with a range of 45 degrees. Mity Safety Exporter® was used to anonymize fundus photographs, which were uploaded to the AI software, RetCAD (version 1.3.1; Thirona, Inc.), and DR scores (DRSs) were quantified on a scale of 0–100.

**Figure 1:**
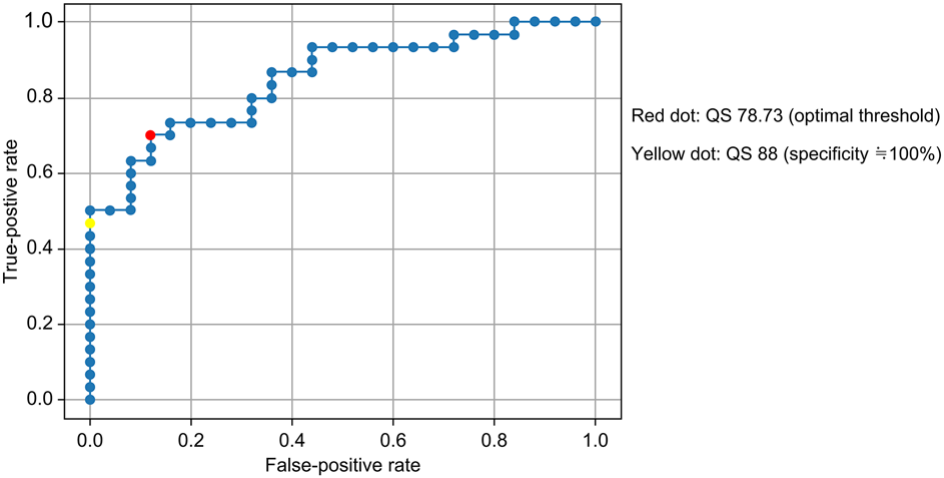
Receiver operating characteristic curves for quality score cutoff value.

A validated cutoff value for the quality score (QS) of fundus images was determined. QS was a score automatically generated by RetCAD. A retinal specialist (K.K.) evaluated 58 fundus images in terms of QS classification. In the ROC curve for setting the QS cutoff value, the AUC was 0.85. The optimal point for sensitivity and specificity is indicated by the red dot, at QS = 78.83 (Fig. 1). To select highly accurate fundus images, the point with a false-positive rate as close to 0 as possible was set and is indicated by the yellow dot, at QS = 88 (Fig. 1). Based on the above results, fundus images with QS > 90 were considered for evaluation.

#### Definition of diabetic stage

DM stage was defined based on treatment history and HbA1c levels as follows: DM0 (no history of DM treatment and HbA1c level < 6.5%), DM1 (no history of DM treatment and HbA1c level ≥ 6.5%), DM2 (history of DM treatment and HbA1c level < 7.5%), and DM3 (history of DM treatment and HbA1c level ≥ 7.5%). HbA1c level criteria were set based on DM diagnosis^25^.

#### Clinical parameters and lifestyle information

The following parameters were evaluated: BMI, blood test results, and vital signs.

Regarding blood tests, the following was assessed: fasting plasma glucose, HbA1c, white blood cell count, red blood cell count, hemoglobin, platelet (Plt) count, aspartate aminotransferase (AST), alanine aminotransferase (ALT), gamma-glutamyl transpeptidase, lactate dehydrogenase, cholinesterase (ChE), total bilirubin (T-Bil), T-Cho, high-density lipoprotein, low-density lipoprotein, triglyceride, uric acid, blood urea nitrogen, blood creatinine, total protein, and albumin. The following vital signs were evaluated: SBP, diastolic blood pressure, and heart rate.

The Fibrosis-4 index (FIB-4), a liver fibrosis index, was calculated using the following formula: FIB-4 = (age × AST)/(Plt count × square root of ALT).

Tobacco smoking and alcohol drinking habits were obtained from Japan’s Questionnaire Sheet for General Health Examinations^26^.

### Study participants

**Figure 2:**
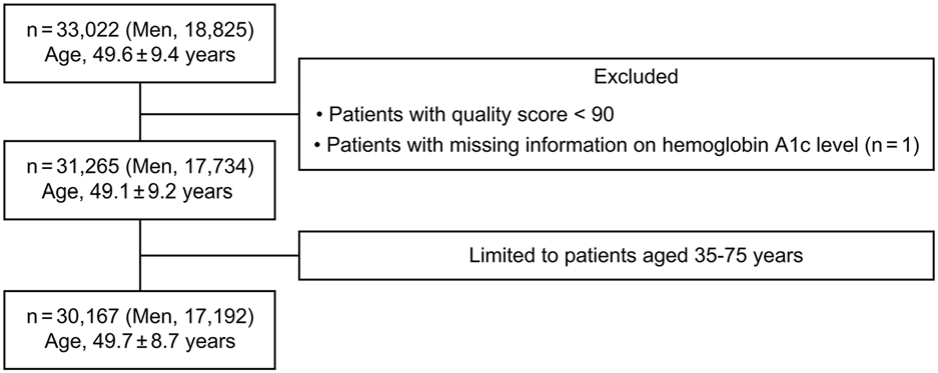
Flow diagram.

Of 33,022 records from both eyes, those with QS < 90 were excluded. For records with the same ID, the record with highest DRS was selected. In total, 30,167 examinees (age range, 35–75 years) were analyzed (Fig. 2).

### Statistical analyses

**Figure 3:**
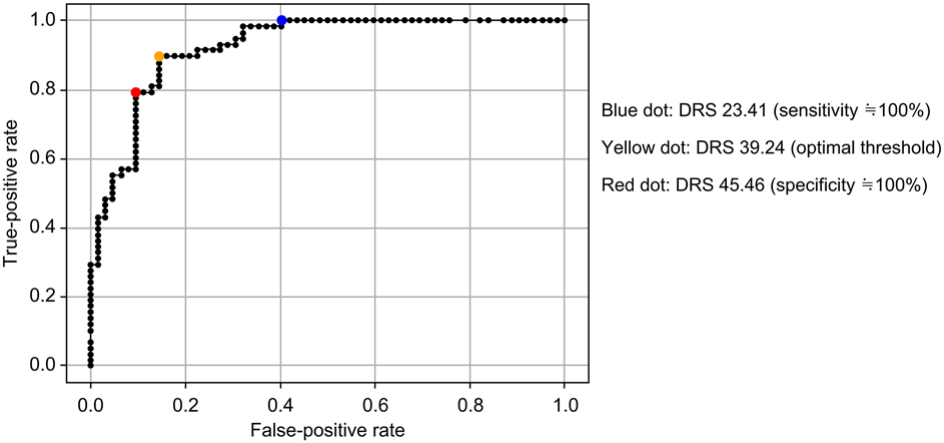
Receiver operating characteristic curves for the diabetic retinopathy score cutoff value.

Pre-validation determined the DRS cutoff value. A retinal specialist (K.K.) evaluated 120 fundus images selected from the analyzed data for DRS classification by reading and grading them on a two-point scale (0, no DR; 1, suspected or probable DR). The cutoff values for the DRS were determined by evaluating the ROC curve (Fig. 3). The AUC was 0.92. The optimal point for sensitivity and specificity is indicated by the yellow dot, at DRS = 39.24 (Fig. 3). The spot at which the sensitivity was as close to 1 as possible in terms of the screening test is indicated by the blue dot, at DRS = 23.14 (Fig. 3). The spot at which the specificity was as close to 1 as possible in the screening test is indicated by the red dot, at DRS = 45.46 (Fig. 3). Based on these results, a well-separated DRS of 20 was defined as the cutoff value for DRS high (with DR) and low (no DR) groups when the sensitivity was approximately 100%, and a DRS of 50 was defined as the cutoff value for the DRS high and low groups when the specificity was approximately 100%.

To determine the association factors of DRS group, examination data of the DRS high and low groups were compared according to DM stage using the analysis of covariance (ANCOVA), with adjustment for age and sex.

Next, to identify factors differing between the DRS high and low groups, logistic analysis was performed with cutoff values of 20 (sensitivity, 100%) and 50 (specificity, 100). Variables were selected based on significant differences by ANCOVA and clinical importance, including SBP, estimated glomerular filtration rate (eGFR), smoking, and urinary protein level.

Finally, to identify the risk factors for DRS, logistic analysis was performed according to DM stage with the cutoff value of 20.

### Informed consent

In this study, informed consent was obtained by opt-out method in accordance with the national legislation and the institutional requirements.

### Supplementary Information

Supplementary Table S1.

## Data Availability

The data are available from the corresponding author upon reasonable request.
