# Supplementary material for: Associated factors of diabetic retinopathy by artificial intelligence evaluation of fundus images in Japan
Source: Sci Rep. 2023 Nov 13;13:19742. doi: 10.1038/s41598-023-47270-x (PMC10643360; doi:10.1038/s41598-023-47270-x)
Supplement: Supplementary file 1 — Supplementary Table S1. [file 41598_2023_47270_MOESM1_ESM.pdf]

Supplemental Table 1. Differences in characteristics stratified by diabetic retinopathy score among diabetes mellitus 1–3 stages

	Variables	DM1 DRS < 20	DM1 DRS ≥ 20	DM1 p-value	DM1 q-value	DM2 DRS < 20	DM2 DRS ≥ 20	DM2 p-value	DM2 q_value	DM3 DRS < 20	DM3 DRS ≥ 20	DM3 p-value	DM3 q-value
Diabetes	**FPG**	134.2 ± 1.5	156.0 ± 6.1	0.001	0.01	124.8 ± 1.0	131.3 ± 2.1	0.003	0.01	171.2 ± 3.1	169.4 ± 5.2	0.75	0.91
	**HbA1c**	7.2 ± 0.1	8.0 ± 0.2	0.000	0.01	6.6 ± 0.0	6.7 ± 0.0	0.13	0.33	8.6 ± 0.1	8.8 ± 0.1	0.10	0.26
Obesity	**BMI**	26.3 ± 0.2	27.0 ± 0.8	0.41	0.82	25.3 ± 0.2	25.2 ± 0.4	0.83	0.93	26.9 ± 0.3	26.3 ± 0.5	0.26	0.38
Vital sign	**SBP**	121.9 ± 0.8	130.6 ± 3.3	0.01	0.08	119.0 ± 0.7	123.9 ± 1.6	0.002	0.01	119.8 ± 1.0	123.0 ± 1.7	0.08	0.22
	**DBP**	76.9 ± 0.6	81.8 ± 2.3	0.04	0.17	73.9 ± 0.5	74.6 ± 1.1	0.50	0.73	74.9 ± 0.7	75.0 ± 1.2	0.99	0.99
	**HR**	76.7 ± 0.6	76.3 ± 2.3	0.86	1.00	74.4 ± 0.5	76.4 ± 1.1	0.09	0.26	77.6 ± 0.8	79.7 ± 1.4	0.15	0.28
Blood cell	**WBC**	6429.0 ± 82.6	6102.7 ± 324.6	0.33	0.70	6112.4 ± 81.5	6335.0 ± 180.1	0.22	0.45	6780.0 ± 138.1	7007.8 ± 230.0	0.35	0.47
	**RBC**	474.5 ± 1.7	474.4 ± 6.8	0.99	1.02	462.4 ± 1.8	453.3 ± 4.0	0.02	0.08	478.0 ± 3.1	476.4 ± 5.2	0.77	0.86
	**Hb**	14.3 ± 0.1	14.3 ± 0.2	0.85	1.04	14.1 ± 0.1	13.8 ± 0.1	0.003	0.05	14.4 ± 0.1	14.3 ± 0.1	0.93	0.96
	**Plt**	25.8 ± 0.3	23.1 ± 1.2	0.02	0.14	23.9 ± 0.2	23.9 ± 0.5	0.95	1.03	24.9 ± 0.4	25.2 ± 0.7	0.76	0.88
Liver function	**AST**	29.2 ± 0.8	30.3 ± 3.0	0.72	0.91	24.7 ± 0.6	24.6 ± 1.3	0.94	0.97	29.1 ± 1.1	23.9 ± 1.9	0.01	0.26
	**ALT**	36.3 ± 1.2	36.5 ± 4.9	0.97	1.04	26.5 ± 0.9	24.2 ± 1.9	0.24	0.51	35.5 ± 1.6	28.3 ± 2.7	0.01	0.16
	**γ-GTP**	55.0 ± 2.8	54.4 ± 11.2	0.95	1.07	40.4 ± 2.0	36.8 ± 4.4	0.43	0.71	55.9 ± 4.8	37.4 ± 8.0	0.03	0.13
	**LDH**	179.8 ± 1.6	182.9 ± 6.2	0.62	0.87	174.4 ± 1.4	174.7 ± 3.2	0.92	0.92	175.3 ± 2.2	175.8 ± 3.6	0.90	0.97
	**ChE**	384.6 ± 3.2	370.5 ± 12.6	0.27	0.70	364.0 ± 3.0	344.2 ± 6.6	0.003	0.02	383.1 ± 4.7	364.9 ± 7.8	0.03	0.16
	**T-Bil**	0.94 ± 0.02	0.94 ± 0.07	0.99	0.99	0.97 ± 0.02	0.84 ± 0.04	0.002	0.01	0.90 ± 0.02	0.86 ± 0.04	0.28	0.39
	**TP**	7.4 ± 0.0	7.3 ± 0.1	0.50	0.78	7.3 ± 0.0	7.3 ± 0.0	0.64	0.81	7.4 ± 0.0	7.3 ± 0.0	0.07	0.21
	**Alb**	4.3 ± 0.0	4.2 ± 0.0	0.16	0.44	4.3 ± 0.0	4.2 ± 0.0	0.005	0.02	4.3 ± 0.0	4.3 ± 0.0	0.11	0.24
	**FIB-4**	1.15 ± 0.02	1.41 ± 0.09	0.01	0.07	1.25 ± 0.03	1.37 ± 0.06	0.06	0.18	1.20 ± 0.04	1.06 ± 0.07	0.05	0.19
Lipid metabolism	**T-cho**	219.6 ± 1.7	205.6 ± 6.7	0.04	0.17	200.0 ± 1.4	197.4 ± 3.0	0.39	0.69	208.5 ± 2.5	199.7 ± 4.2	0.05	0.18
	**HDL**	58.6 ± 0.7	56.4 ± 2.6	0.42	0.78	62.9 ± 0.7	62.1 ± 1.6	0.59	0.69	58.0 ± 1.1	60.3 ± 1.8	0.24	0.39
	**LDL**	137.0 ± 1.5	127.3 ± 5.9	0.11	0.38	118.0 ± 1.2	116.9 ± 2.7	0.69	0.84	125.6 ± 2.1	119.6 ± 3.5	0.11	0.25
	**TG**	144.0 ± 6.1	124.2 ± 23.8	0.42	0.73	113.6 ± 3.5	109.6 ± 7.8	0.61	0.85	152.9 ± 8.3	117.0 ± 13.8	0.01	0.13
Renal function	**BUN**	13.1 ± 0.2	12.7 ± 0.6	0.56	0.83	13.7 ± 0.2	14.1 ± 0.4	0.32	0.60	13.5 ± 0.2	13.2 ± 0.4	0.50	0.64
	**UA**	5.4 ± 0.1	5.7 ± 0.2	0.31	0.72	5.4 ± 0.1	5.4 ± 0.1	0.71	0.95	5.0 ± 0.1	4.7 ± 0.1	0.12	0.24
	**Cre**	0.75 ± 0.01	0.72 ± 0.05	0.64	0.86	0.75 ± 0.02	0.92 ± 0.05	0.00	0.02	0.71 ± 0.03	0.76 ± 0.04	0.25	0.39
	**eGFR**	77.6 ± 0.7	81.9 ± 2.8	0.13	0.41	74.8 ± 0.7	73.7 ± 1.5	0.47	0.72	81.6 ± 1.1	84.5 ± 1.9	0.15	0.26
Inflammation	**CRP**	0.25 ± 0.03	0.33 ± 0.10	0.47	0.78	0.16 ± 0.02	0.23 ± 0.05	0.18	0.43	0.30 ± 0.04	0.14 ± 0.07	0.02	0.17
Categorical data	**Sex, men**	383 (72.3%)	19 (67.9%)	0.62		597 (80.3%)	96 (86.5%)	0.11		235 (78.1%)	79 (84.9%)	0.14	
	**Urinary protein, yes**	18 (3.4%)	2 (7.1%)	0.35		26 (3.5%)	10 (9.0%)	0.02		27 (9.0%)	12 (12.9%)	0.28	
	**Smoking, yes**	154 (29.1%)	8 (28.6%)	0.96		193 (26.0%)	32 (28.8%)	0.53		94 (31.2%)	42 (45.2%)	0.01	
	**Drinking, yes**	325 (61.3%)	19 (67.9%)	0.75		498 (67.0%)	72 (64.9%)	0.65		184 (61.1%)	50 (53.8%)	0.21	

Abbreviations: DM = diabetes mellitus, DRS = diabetic retinopathy score, FPG = fasting plasma glucose, Hb = hemoglobin, BMI = body mass index, SBP = systolic blood pressure, DBP = diastolic blood pressure, HR = heart rate, WBC = white blood cell, RBC = red blood cell, Plt = platelet, AST = aspartate aminotransferase, ALT = alanine aminotransferase, γ-GTP = gamma-glutamyl transpeptidase, LDH = lactate dehydrogenase, ChE = cholinesterase, T-Bil = total bilirubin, TP = total protein, Alb = albumin, FIB-4 = fibrosis-4 index, T-Cho = total cholesterol, HDL = high-density lipoprotein, LDL = low-density lipoprotein, TG = triglyceride, BUN = blood urea nitrogen, UA = uric acid, Cre = blood creatinine, eGFR = estimated glomerular filtration rate, CRP = C-reactive protein.

The FIB-4 index score was calculated using the following formula. FIB-4 = (age × AST)/(Plt count × square root of ALT).

Continuous variables are presented as mean ± standard error by analysis of covariance after adjusting for age and sex. Categorical variables are presented as n (%), and p-values are calculated using the chi-squared test.

The q-values for continuous variables are calculated by modifying the p-value using the false discovery rate method.
